# Pulpotomy in Primary Molars with Deep Caries Using Biodentine: A Case Series with 1-Year Follow-Up

**DOI:** 10.3390/dj14080521

**Published:** 2026-08-14

**Authors:** Shiya Wang, Qiong Zhang, Ran Yang, Jing Zou, Yan Wang

**Affiliations:** 1 State Key Laboratory of Oral Diseases & National Center for Stomatology & National Clinical Research Center for Oral Diseases, Department of Pediatric Dentistry, West China Hospital of Stomatology, Sichuan University, Chengdu 610041, China; wang_sya@163.com (S.W.); qiongzhang@scu.edu.cn (Q.Z.); yangran@scu.edu.cn (R.Y.); 2 Department of West Outpatient, West China Hospital of Stomatology, Sichuan University, Chengdu 610031, China

**Keywords:** deep caries, pulpotomy, stainless steel crown, Biodentine, case series

## Abstract

**Background**: Biodentine has emerged as a promising calcium silicate-based material for vital pulp therapy in primary teeth. This case series describes short-term clinical and radiographic outcomes of Biodentine pulpotomy followed by immediate stainless steel crown restoration for deeply carious primary molars treated under general anesthesia. **Methods**: Data were collected prospectively within an ongoing clinical project. Five pediatric patients, aged 3 to 6 years, presented with multiple carious primary teeth. A total of 15 deeply carious primary molars without clinical or radiographic signs of irreversible pulpitis, pulp necrosis, or periradicular pathology were included. Under general anesthesia, pulpotomies were performed. Pulp status was assessed intraoperatively based on bright-red pulp bleeding and hemostasis achieved within 5 min. Biodentine was applied to cover the pulp stumps and fill the cavities, followed immediately by stainless steel crown restoration. **Results:** During the 1-year follow-up, all treated teeth maintained normal chewing function without unfavorable symptoms. Radiographic examination revealed normal periodontal ligament spaces and no furcation or periapical radiolucency, pathological internal or external root resorption, or abnormality of the developing permanent tooth germs. **Conclusions:** For deeply carious primary molars, Biodentine pulpotomy followed by immediate stainless steel crown restoration showed favorable short-term clinical and radiographic outcomes. This single-session approach may represent a feasible option for deeply carious primary molars.

## 1. Introduction

Dental caries in the primary dentition remains a major childhood oral health problem [1]. According to the 2022 Global Oral Health Status Report, untreated caries in primary teeth affects approximately 514 million children worldwide, with an estimated prevalence of 43.1% [2]. Dental caries in primary teeth may compromise essential oral functions, including mastication, speech, and aesthetics, and may further affect dietary intake, sleep, facial appearance, self-esteem, and psychosocial well-being, thereby reducing the oral health-related quality of life of both children and their families [3,4]. Because primary molars have relatively thin enamel and dentin and comparatively large pulp chambers, carious lesions may progress rapidly toward the pulp if not managed in time. Advanced lesions may result in pulp inflammation, odontogenic infection, premature tooth loss, space loss, and potential disturbance of the developing permanent successors [5,6]. Therefore, timely and appropriate management of carious primary teeth until physiological exfoliation remains an important goal in pediatric dentistry.

Deep caries approaching the pulp presents a difficult treatment decision. For vital primary molars without clinical or radiographic signs of irreversible pulpitis, pulp necrosis, furcation involvement, or pathological root resorption, vital pulp therapy (VPT) is recommended to maintain radicular pulp vitality and tooth function [7]. Pulpotomy is a conservative VPT that involves removal of the infected coronal pulp, preservation of the radicular pulp, placement of a biocompatible material over the pulp stumps, and restoration with an adequate coronal seal [8]. The main objectives of VPT are to keep teeth symptomless and functional until their physiological exfoliation [9]. Compared to pulpectomy, pulpotomy is less traumatic and more time-efficient. These advantages are particularly relevant in young children and in comprehensive dental treatment under general anesthesia, where procedural efficiency and long-term tooth retention are important considerations [10]. With appropriate case selection, pulpotomy has been shown to achieve favorable clinical and radiographic outcomes comparable to those of pulpectomy in primary molars [8,11].

The prognosis of pulpotomy depends on accurate diagnosis, intraoperative assessment of pulp status, the pulp-covering material, and the quality of the final coronal restoration. Various medicaments have been used for pulpotomy in primary teeth, including formocresol, ferric sulfate, calcium hydroxide, mineral trioxide aggregate, and other calcium silicate-based materials [12]. Although formocresol has historically been used as a conventional pulpotomy medicament, concerns have been raised regarding its potential cytotoxicity, mutagenicity and carcinogenicity, and damage to permanent teeth [13,14]. These concerns have encouraged the development and clinical application of more biocompatible and bioactive alternatives. In recent years, calcium silicate cements have gained increasing attention because of their favorable biocompatibility, sealing ability, and capacity to promote hard tissue repair. Biodentine, a tricalcium silicate-based material, has been introduced as an alternative to mineral trioxide aggregate, with advantages including good biocompatibility, dentin-like mechanical properties, bioactivity, and a relatively short setting time [15]. However, even when a suitable pulp medicament is used, coronal microleakage may compromise the long-term outcome [16]. The stainless steel crowns (SSCs) provide full-coronal coverage and durable restoration for extensively carious primary molars, and their effective coronal seal may reduce microleakage and bacterial reinfection [17].

Based on the above considerations, the present case series describes the 1-year clinical and radiographic outcomes of Biodentine pulpotomy followed by immediate SSC restoration in deeply carious primary molars treated under general anesthesia. This report may provide useful clinical insight into the application of pulpotomy for preserving deeply carious primary molars in pediatric patients.

## 2. Case Presentation

### 2.1. Study Design, Case Selection, and Reporting

Data for this descriptive case series were collected within an ongoing prospective clinical project conducted at the Department of Pediatric Dentistry, West China Hospital of Stomatology, Sichuan University. The present report describes five non-consecutive patients treated between 22 May and 26 June 2024. Final follow-up assessments were completed between 22 June and 6 September 2025; therefore, the reporting period extended from 22 May 2024 to 6 September 2025. The five patients were included because complete and verifiable clinical and radiographic records were available through a final follow-up assessment conducted at least 1 year after treatment. Additional patients enrolled in the clinical project were not included in this report. Because the reported cases were not consecutive, selection bias cannot be excluded. This manuscript was prepared in accordance with the CARE reporting guidelines, as applicable to a multiple-patient case series [18].

### 2.2. Inclusion and Exclusion Criteria

The inclusion criteria were as follows: (1) the children were systemically healthy at the time of treatment and throughout the follow-up period; (2) the treated tooth was a primary molar diagnosed with deep caries or chronic pulpitis clinically judged to be confined to the coronal pulp, without congenital or developmental dental anomalies; (3) the child had no persistent spontaneous pain or nocturnal pain associated with the treated tooth; (4) clinical examination revealed no pathological mobility, gingival swelling, sinus tract, or abscess; (5) preoperative radiographs showed caries extending into deep dentin or approaching the pulp, without evident pathological root resorption, furcation or periapical radiolucency, or abnormal widening of the periodontal ligament space, and showed a normally developing permanent successor with intact surrounding bone; and (6) the parents or legal guardians had received an explanation of the clinical project, agreed to their child’s participation, and provided written informed consent. For inclusion in this report, complete clinical and radiographic documentation through final follow-up was also required.

The exclusion criteria were persistent spontaneous pain, nocturnal pain, or other clinical signs or symptoms suggestive of irreversible pulpitis, as well as radiographic evidence of marked or pathological root resorption, furcation or periapical radiolucency, internal root resorption, or pulp calcification.

### 2.3. Patient Information and Diagnostic Assessment

Five pediatric patients, including three boys and two girls aged 3 to 6 years, presented to the Department of Pediatric Dentistry, West China Hospital of Stomatology, Sichuan University, with a chief complaint of multiple carious primary teeth. All patients had a history of prolonged nocturnal milk feeding. None of the patients reported any discomfort associated with their teeth, such as spontaneous pain or sensitivity to thermal stimuli.

Clinical examination showed poor oral hygiene in all patients, who were in either the primary or early mixed dentition stage. According to the the Fédération Dentaire Internationale two-digit tooth notation system, the following 15 primary molars met the eligibility criteria: teeth 84 and 85 in Patient 1; teeth 55, 74, and 75 in Patient 2; teeth 54, 84, and 85 in Patient 3; teeth 54, 55, and 84 in Patient 4; and teeth 54, 55, 84, and 85 in Patient 5. The lesions mainly involved the occlusal, mesial, and buccal surfaces. No abnormal tooth mobility, gingival swelling, sinus tract, abscess, or oral mucosal lesion was observed. Radiographs showed radiolucent lesions extending close to the pulp chamber, without definitive signs of periapical or furcation radiolucency, pathological root resorption, or abnormality of the developing permanent tooth germs.

Based on the clinical and radiographic findings, the included teeth were diagnosed with deep caries or pulp inflammation considered clinically confined to the coronal pulp and were judged suitable for pulpotomy. Given the young age of the patients, their limited ability to cooperate with extensive treatment, the presence of multiple carious teeth, and the need for comprehensive dental care, treatment under general anesthesia was selected. The 15 included teeth underwent pulpotomy followed by immediate SSC restoration. Other teeth were treated according to their individual diagnoses during the same comprehensive treatment sessions but were not included in this case series. The treatment procedure, potential benefits, and possible risks were explained in detail to the parents or legal guardians, and written informed consent was obtained before treatment.

### 2.4. Therapeutic Intervention

**Figure 1:**
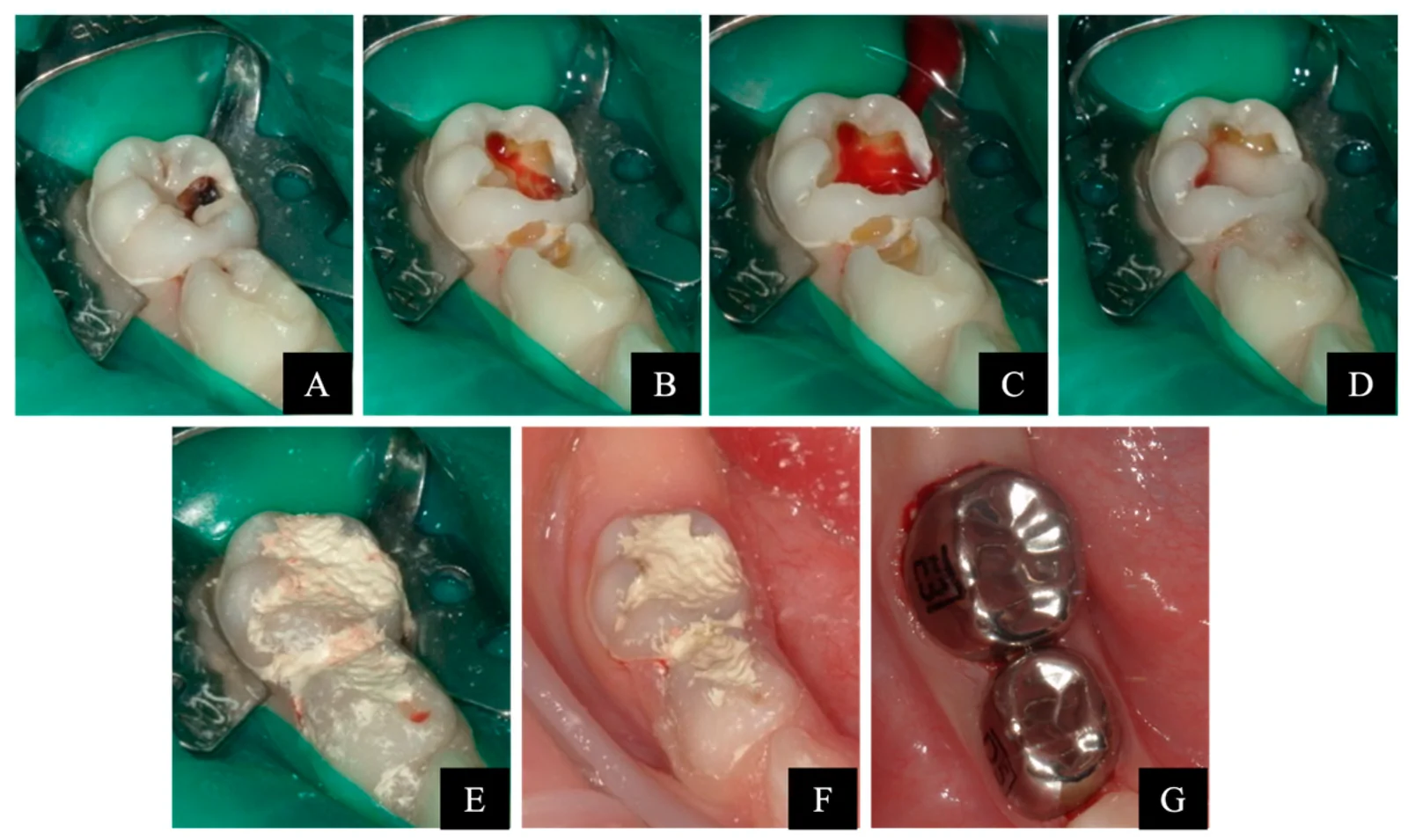
Clinical steps of pulpotomy and SSC restoration. (**A**) Rubber dam isolation; (**B**) Removal of decayed tissue, exposing the pulp and performing pulp chamber access; (**C**) Amputation of the coronal pulp; (**D**) Hemostasis achieved using a saline-soaked cotton pellet applied to the radicular pulp stumps; (**E**) Placement of Biodentine to cover the pulp stumps and simultaneously fill the cavity; (**F**) Removal of rubber dam after initial setting of Biodentine; (**G**) Final restoration with a cemented SSC.

All five patients underwent general anesthesia with nasotracheal intubation, which maintained unobstructed oral access and permitted rubber dam isolation to establish a clean and dry operating field (Figure 1A). Caries excavation was performed using a sterile round diamond bur. Deep carious dentin close to the pulp was carefully removed using a hand excavator and a slow-speed round bur. After pulp exposure was confirmed, the roof of the pulp chamber was removed using a sterile round diamond bur in a high-speed handpiece (Figure 1B). Pulp vitality was assessed intraoperatively based on the presence of bright red bleeding from the pulp tissue. Under a dental operating microscope (Zumax Medical Co., Ltd., Suzhou, China), the affected coronal pulp was carefully amputated (Figure 1C). The pulp chamber was irrigated alternately with 1% sodium hypochlorite solution (Longly Biotechnology (Wuhan) Co., Ltd., Wuhan, China) and 0.9% sterile saline (Sodium Chloride Injection; Sichuan Kelun Pharmaceutical Co., Ltd., Chengdu, China), after which bleeding from the radicular pulp stumps was evaluated. Hemostasis was achieved by applying gentle pressure with a sterile cotton pellet moistened with sterile saline for 5 min (Figure 1D). Successful hemostasis within 5 min was considered indicative of a favorable radicular pulp status, and the pulpotomy procedure was continued. Following hemostasis, Biodentine (Septodont, Saint-Maur-des-Fossés, France) was mixed according to the manufacturer’s instructions and placed into the pulp chamber to cover the radicular pulp stumps and simultaneously fill the cavity (Figure 1E). After setting, an appropriately sized SSC was selected from either the Kids Crown system (Shinhung Co., Ltd., Seoul, Republic of Korea) or the 3M ESPE Stainless Steel Primary Molar Crown system (3M ESPE, St. Paul, MN, USA), based on the morphology and dimensions of the treated primary tooth. The crown was cemented with glass ionomer luting cement (GC Fuji I; GC Dental (Suzhou) Co., Ltd., Suzhou, China) (Figure 1F,G).

### 2.5. Outcomes Assessment and Follow-Up

The primary outcome was overall tooth-level treatment success at the final follow-up assessment, which was conducted at least 1 year after treatment. A treated tooth was considered successful only when both the clinical and radiographic criteria were met. Clinical success required the tooth to remain asymptomatic and functional, with no spontaneous or nocturnal pain, tenderness to percussion, pathological mobility, swelling, sinus tract, or abscess. Radiographic success required the absence of newly developed furcation or periapical radiolucency, pathological internal or external root resorption, abnormal periodontal ligament changes, and adverse effects on the developing permanent successor. Physiological root resorption consistent with the child’s developmental stage was regarded as a normal finding rather than treatment failure. A tooth was classified as having failed if any clinical or radiographic failure criterion was present or if unplanned pulp retreatment or extraction related to the treated tooth was required.

Clinical assessments were performed at approximately 1 and 6 months and at the final follow-up, whereas radiographic assessments were performed preoperatively, at approximately 6 months, and at the final follow-up. Two pediatric dentists (Shiya Wang and Yan Wang) independently reviewed the clinical follow-up records and radiographs according to the predefined criteria. Before assessment, the evaluators reviewed and agreed upon the operational definitions. Any disagreement was resolved through discussion and consensus. The patient-level timeline is presented in Table 1.

**Figure 2:**
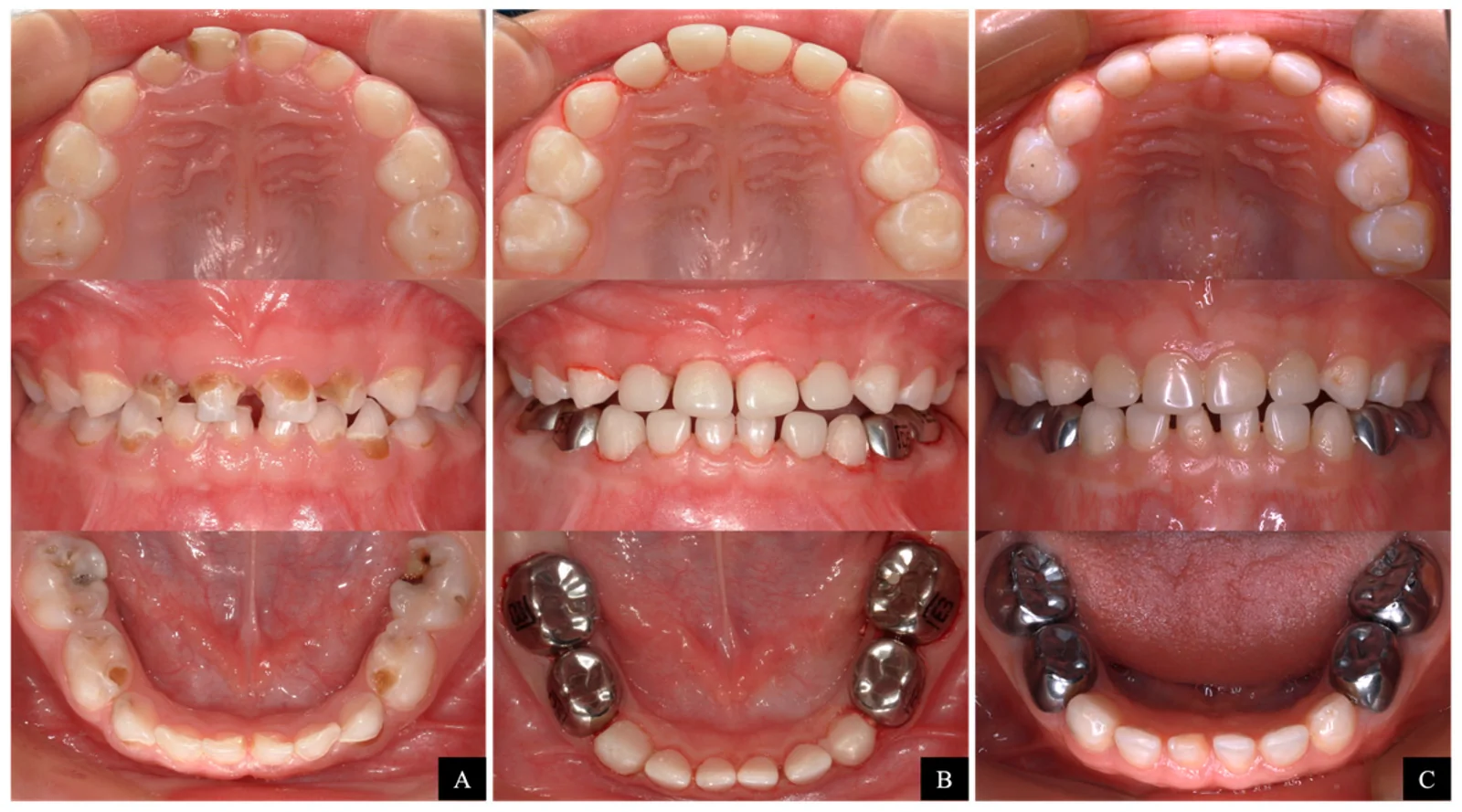
Intraoral clinical photographs of Patient 1. (**A**) Preoperative view; (**B**) Immediate postoperative view; (**C**) Final follow-up view.

**Figure 3:**
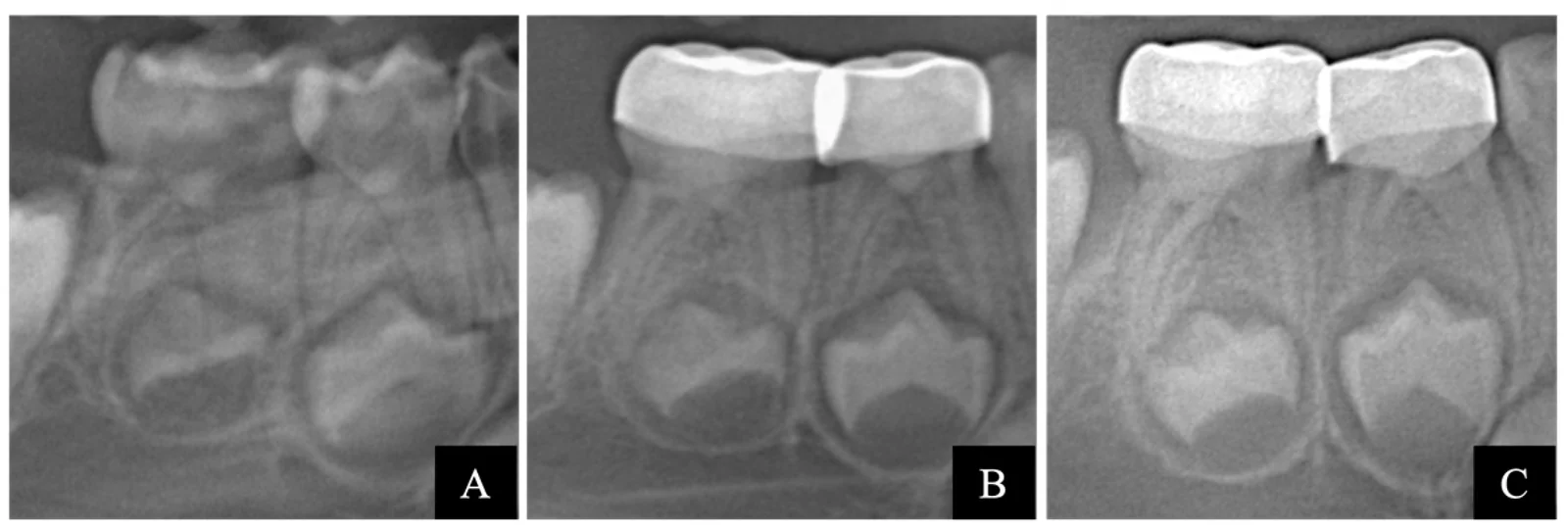
Radiographic evaluation of teeth 84 and 85 in Patient 1. (**A**) Preoperative radiograph; (**B**) 6-month post-treatment radiograph; (**C**) final follow-up radiograph.

**Figure 4:**
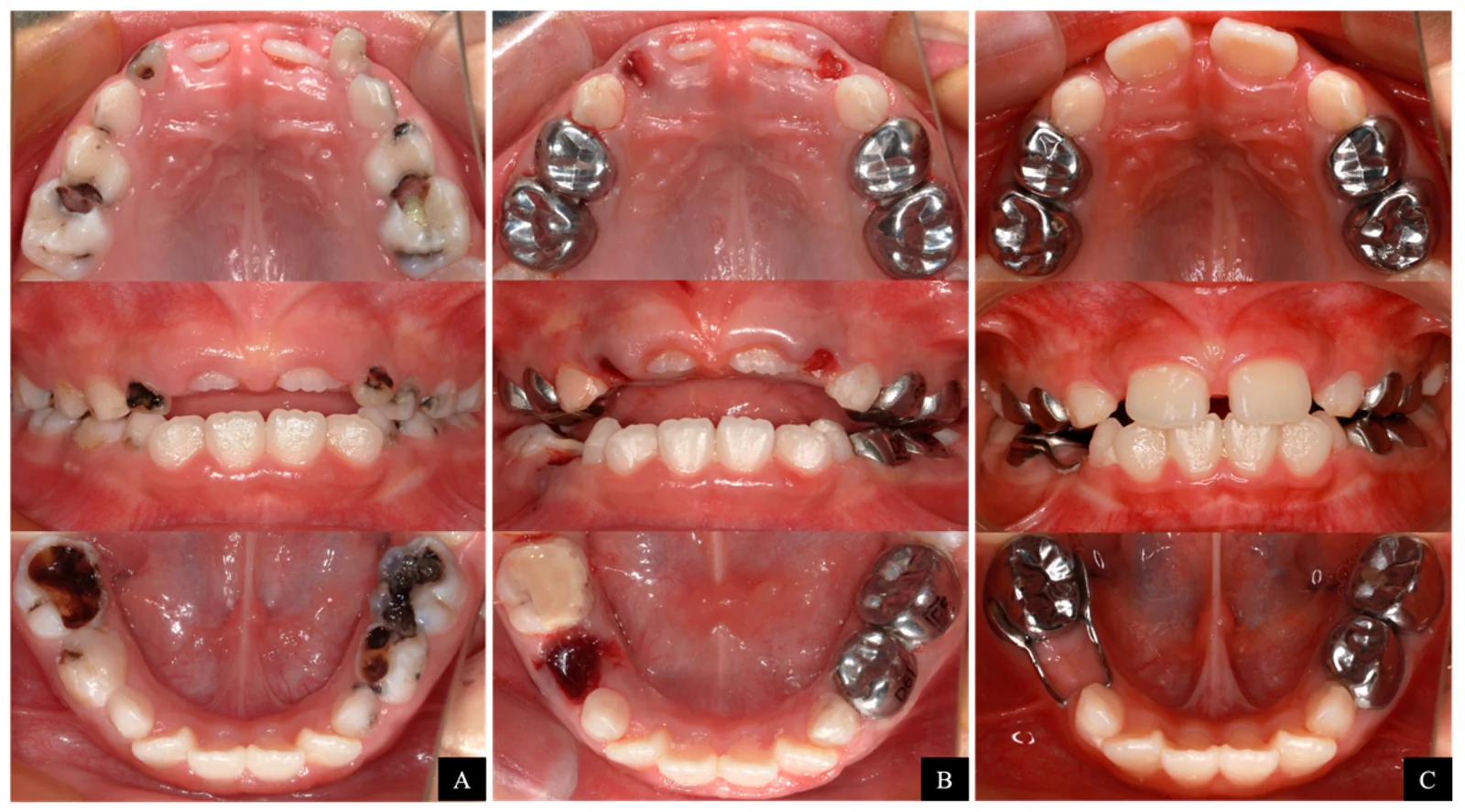
Intraoral clinical photographs of Patient 2. (**A**) Preoperative view; (**B**) Immediate postoperative view; (**C**) Final follow-up view. The crown-and-loop space maintainer shown in (**C**), with tooth 85 serving as the abutment, was fitted and cemented at a 1-week postoperative outpatient visit while the patient was awake. Tooth 85 and the space-maintenance procedure were not included in the present outcome assessment.

**Figure 5:**
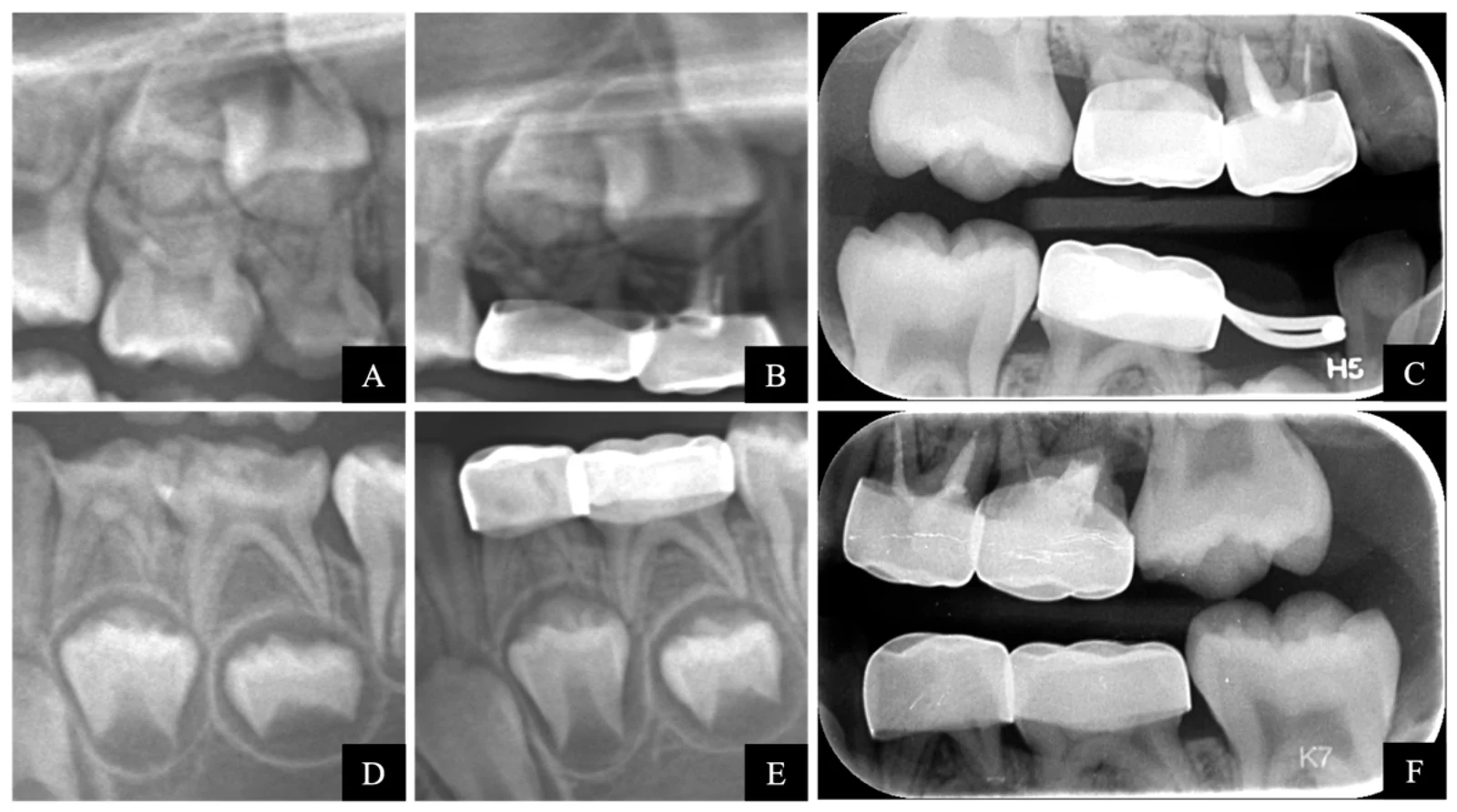
Radiographic evaluation of teeth 55, 74, and 75 in Patient 2. (**A**–**C**) Tooth 55: (**A**) Preoperative radiograph; (**B**) 6-month post-treatment radiograph; (**C**) final follow-up bitewing radiograph. (**D**–**F**) Teeth 74 and 75: (**D**) Preoperative radiograph; (**E**) 6-month post-treatment radiograph; (**F**) final follow-up bitewing radiograph. Teeth 54, 64, and 65 which are visible in the final bitewing views had undergone pulpectomy. These teeth were not included in the present outcome assessment.

**Figure 6:**
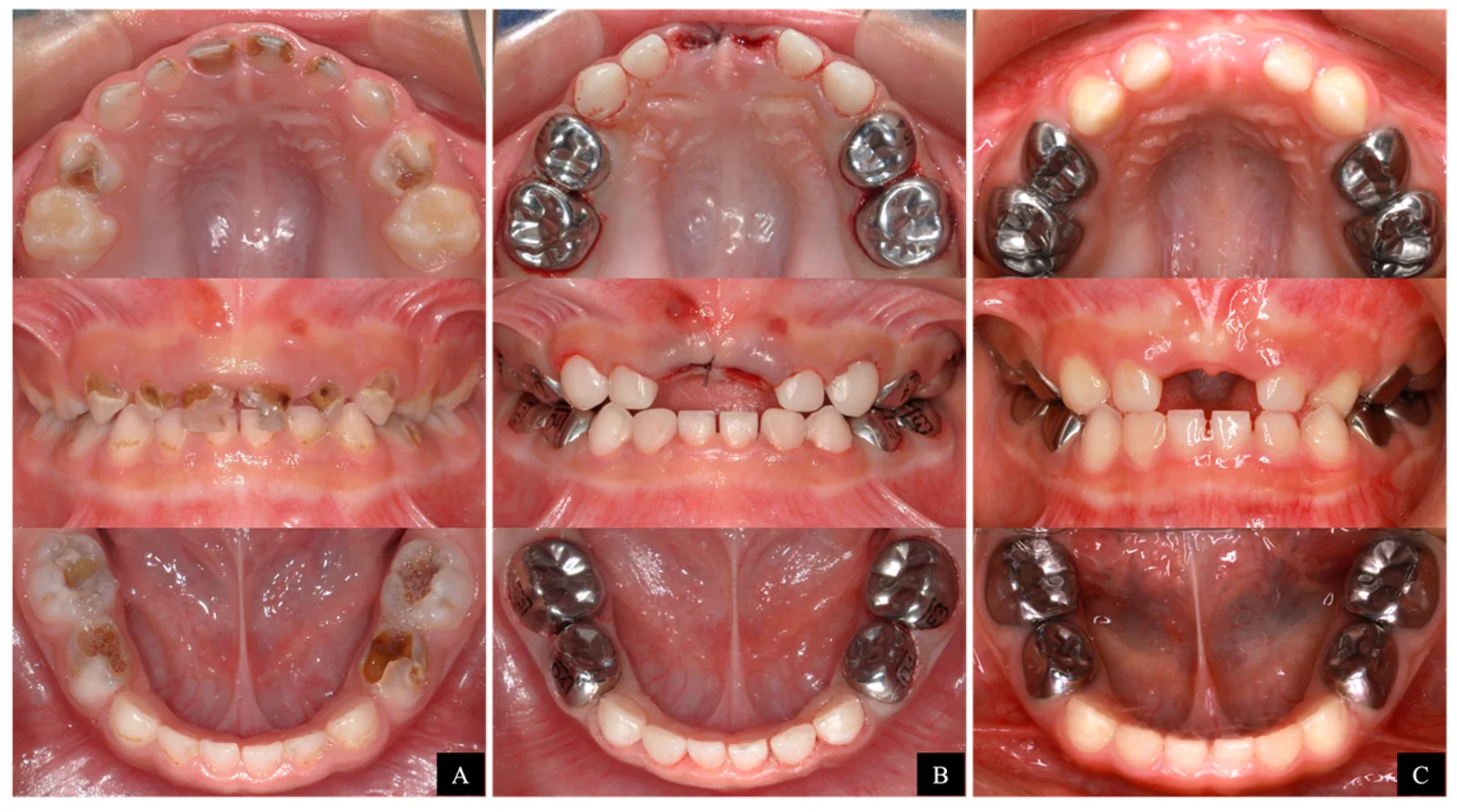
Intraoral clinical photographs of Patient 3. (**A**) Preoperative view; (**B**) Immediate postoperative view; (**C**) Final follow-up view.

**Figure 7:**
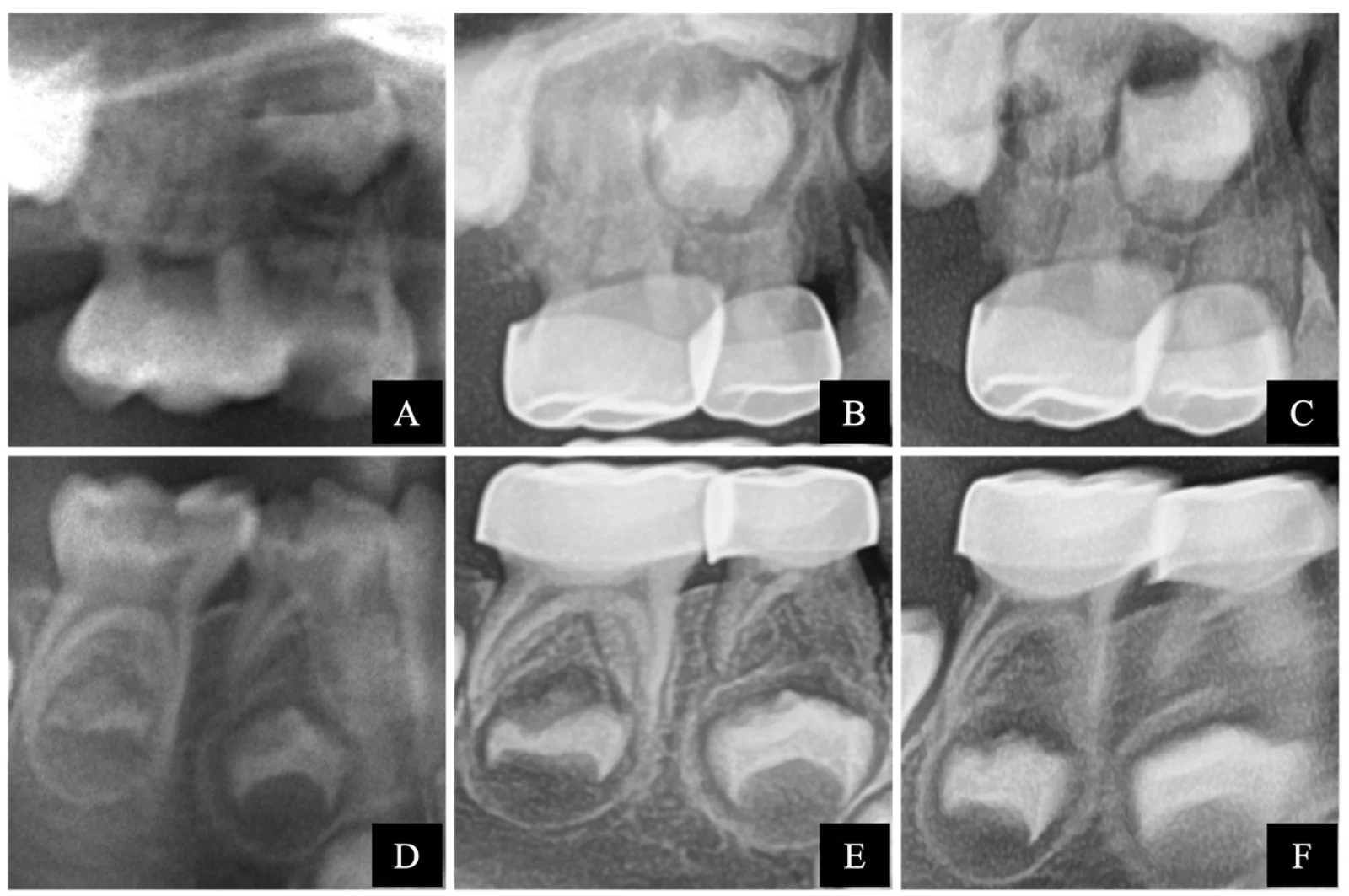
Radiographic evaluation of teeth 54, 84, and 85 in Patient 3. (**A**–**C**) Tooth 54: (**A**) Preoperative radiograph; (**B**) 6-month post-treatment radiograph; (**C**) final follow-up radiograph. (**D**–**F**) Teeth 84 and 85: (**D**) Preoperative radiograph; (**E**) 6-month post-treatment radiograph; (**F**) final follow-up radiograph.

**Figure 8:**
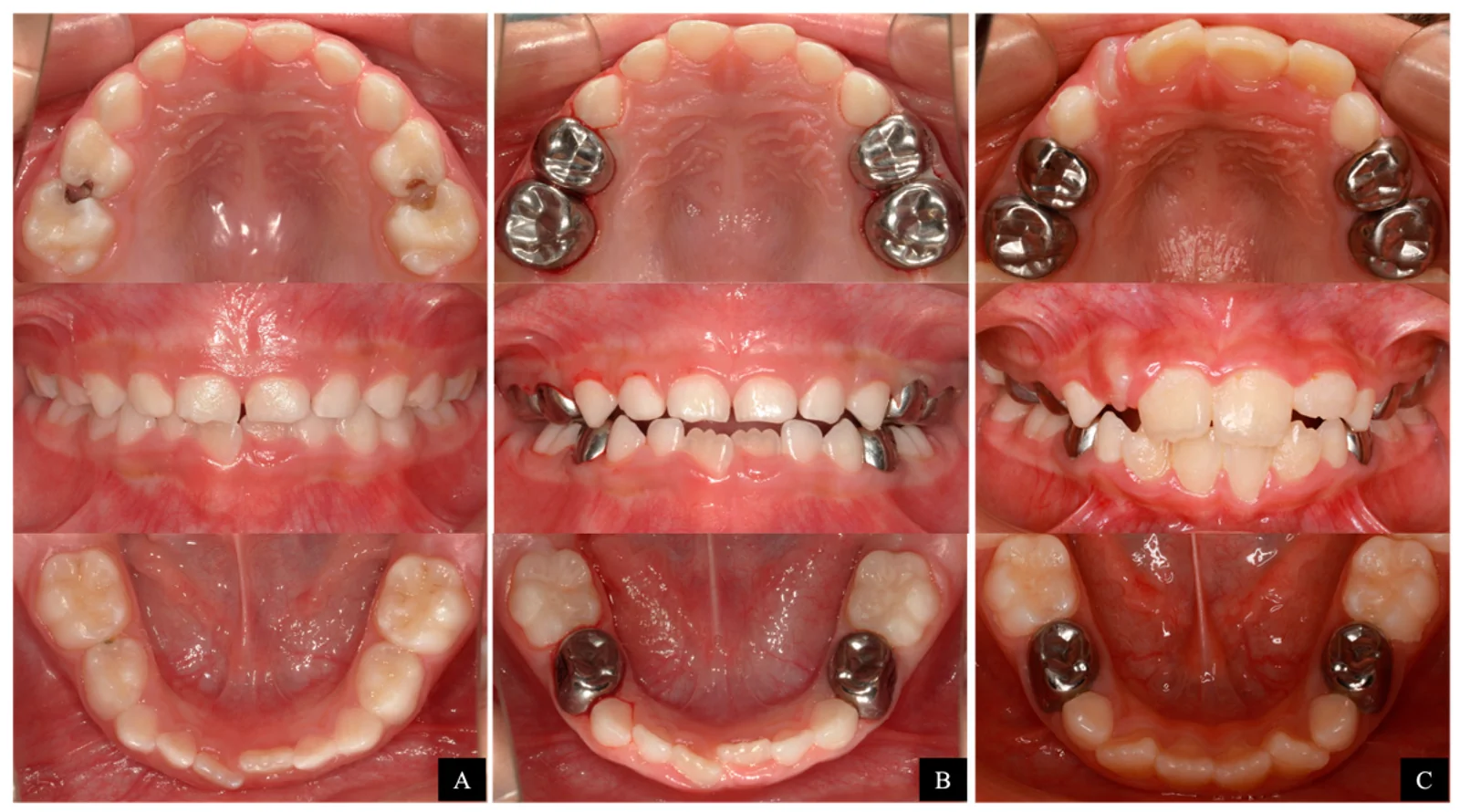
Intraoral clinical photographs of Patient 4. (**A**) Preoperative view; (**B**) Immediate postoperative view; (**C**) Final follow-up view.

**Figure 9:**
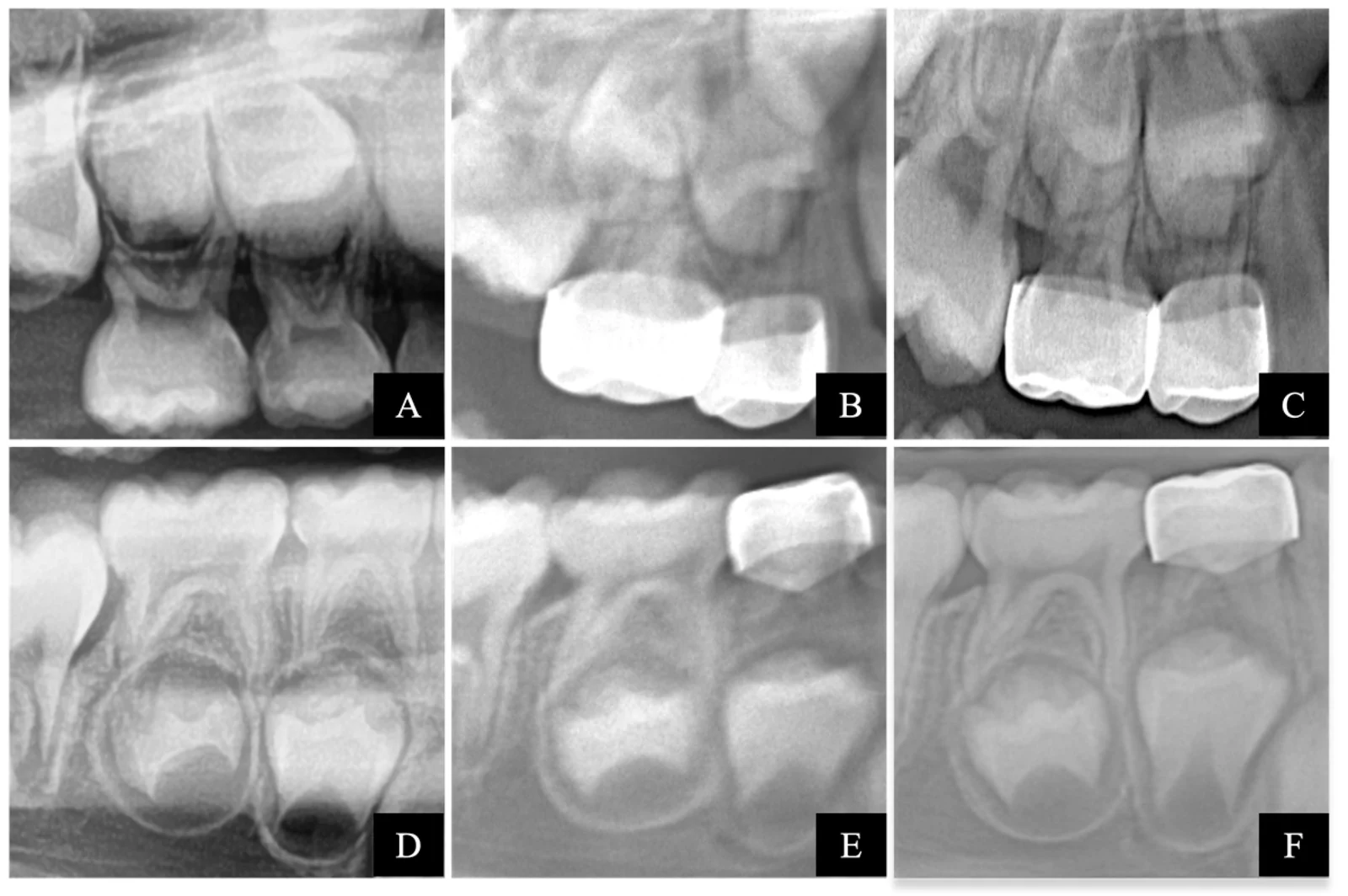
Radiographic evaluation of teeth 54, 55, and 84 in Patient 4. (**A**–**C**) Teeth 54 and 55: (**A**) Preoperative radiograph; (**B**) 6-month post-treatment radiograph; (**C**) final follow-up radiograph. (**D**–**F**) Tooth 84: (**D**) Preoperative radiograph; (**E**) 6-month post-treatment radiograph; (**F**) final follow-up radiograph.

**Figure 10:**
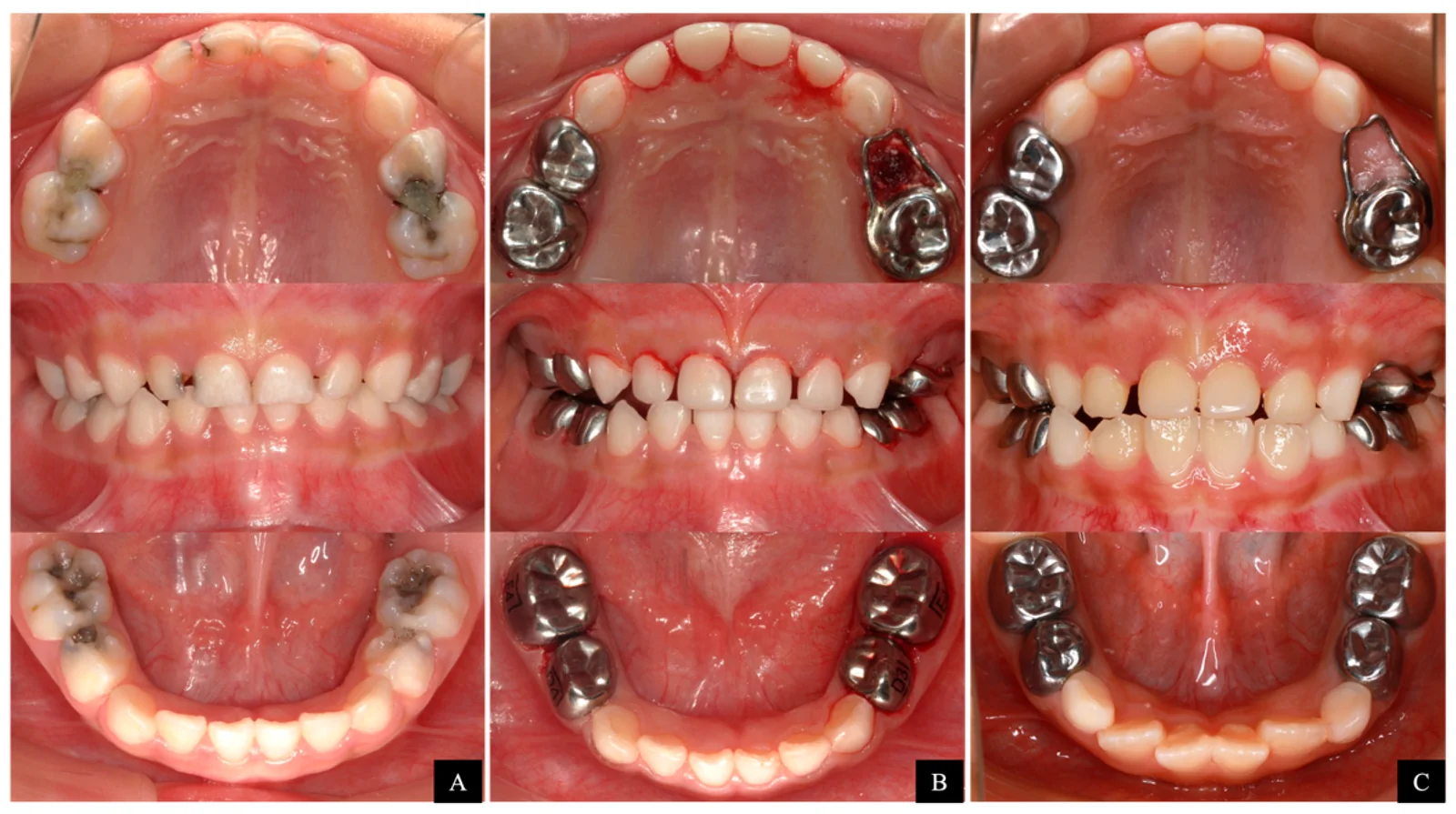
Intraoral clinical photographs of Patient 5. (**A**) Preoperative view; (**B**) Immediate postoperative view. The crown-and-loop space maintainer shown in (**B**), with tooth 65 serving as the abutment, was fabricated and placed while the patient was under general anesthesia. (**C**) Final follow-up view. Tooth 65 and the space-maintenance procedure were not included in the present outcome assessment.

**Figure 11:**
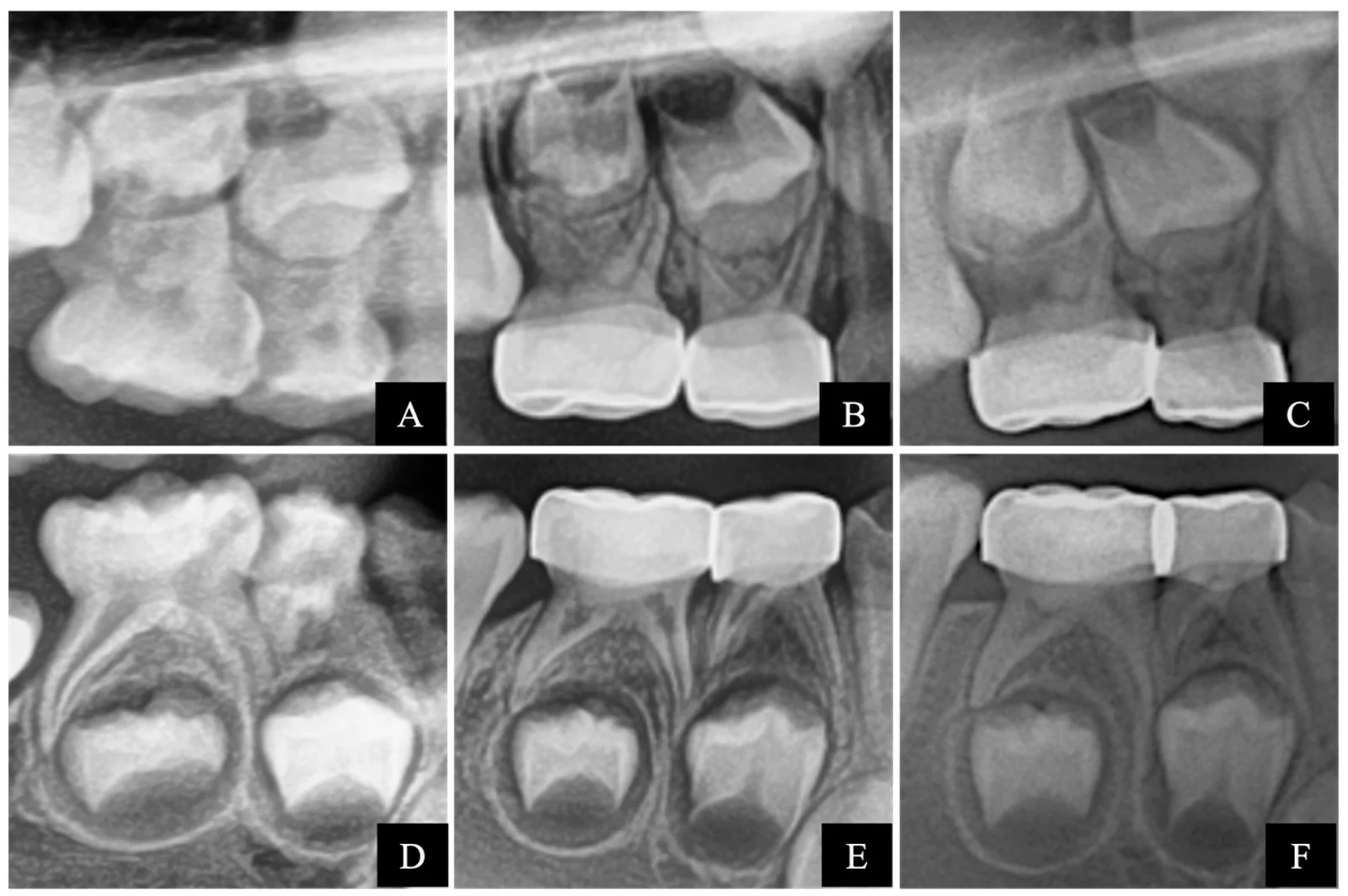
Radiographic evaluation of teeth 54, 55, 84 and 85 in Patient 5. (**A**–**C**) Teeth 54 and 55: (**A**) Preoperative radiograph; (**B**) 6-month post-treatment radiograph; (**C**) final follow-up radiograph. (**D**–**F**) Teeth 84 and 85: (**D**) Preoperative radiograph; (**E**) 6-month post-treatment radiograph; (**F**) final follow-up radiograph.

All patients completed the scheduled follow-up assessments, and no patient was lost to follow-up. Representative intraoral clinical photographs and radiographs for each patient are shown in Figure 2, Figure 3, Figure 4, Figure 5, Figure 6, Figure 7, Figure 8, Figure 9, Figure 10 and Figure 11. At the final follow-up, all treated teeth met both the predefined clinical and radiographic success criteria. The teeth remained asymptomatic, with no spontaneous or nocturnal pain. Normal masticatory function was maintained. The SSCs remained intact and well adapted. All treated teeth showed negative responses to percussion, no pathological mobility, and healthy surrounding soft tissues without swelling, sinus tract, or abscess formation. Radiographically, the furcation and periapical regions showed normal bone architecture without radiolucency. The periodontal ligament spaces were within normal limits, and no pathological internal or external root resorption was detected. Notably, radiopaque zones were observed beneath the capping interface in several cases, suggestive of hard tissue deposition, possibly corresponding to reparative dentin bridge formation. Any root resorption observed during follow-up was considered physiological and consistent with the patients’ developmental stage. The underlying permanent tooth germs showed normal development. No treatment-related adverse or unanticipated events were observed.

## 3. Discussion

The present case series describes the short-term clinical and radiographic outcomes of Biodentine pulpotomy followed by immediate SSC restoration in deeply carious primary molars treated under general anesthesia. At the final follow-up, all 15 treated teeth with Biodentine pulpotomy and immediate SSC restoration remained asymptomatic and functional, without clinical or radiographic signs of treatment failure. These observations indicate favorable short-term outcomes of the combined protocol in the reported cases.

The management of deep caries with pulp involvement in primary teeth remains a clinical challenge. Traditionally, it has been assumed that once caries encroaches upon the pulp in young children, the resulting inflammation typically progresses in a diffuse manner, which raises concerns about the prognosis of pulpotomy. As a result, pulpectomy has often been preferred as a relatively “secure” treatment option [19]. However, a deeper understanding of primary pulp pathological changes and accumulating evidence suggest that pulpotomy can yield stable, predictable outcomes [7,20,21]. Clinical evidence suggests that in diagnostically borderline primary molars in which clinical and radiographic findings do not clearly distinguish reversible from irreversible pulpitis, pulpotomy may achieve favorable or even superior long-term survival compared with pulpectomy [11].

Careful preoperative and intraoperative assessment was essential for appropriate case selection. None of the included teeth presented with clinical or radiographic signs suggestive of irreversible pulpitis or apical periodontitis. Nevertheless, clinical and radiographic findings alone are often insufficient to precisely reflect the histological status of the pulp in primary teeth. This diagnostic uncertainty becomes even more relevant during pulpotomy under general anesthesia, because patients are unable to provide real-time subjective feedback regarding pain. Therefore, clinicians must rely more heavily on objective intraoperative indicators. Although previous studies have explored parameters such as caries color, lesion extent, and bleeding characteristics to estimate the pulp inflammation, universally accepted criteria for intraoperative pulp assessment have not yet been established [7,22,23]. In this case series, the nature of pulp bleeding and the duration required to achieve hemostasis were used as the main intraoperative indicators. In all treated teeth, the amputated pulp stumps exhibited bright-red bleeding, and hemostasis was consistently achieved within 5 min using saline-moistened cotton pellets. The absence of clinical and radiographic failure during follow-up suggests that these two bleeding-related parameters may serve as practical indicators for assessing radicular pulp status. However, they should not be regarded as independent diagnostic criteria and must be interpreted in combination with preoperative symptoms and radiographic findings.

The favorable outcomes observed in this case series may also be related to the biological and physicochemical properties of Biodentine. Biodentine is a tricalcium silicate-based cement composed primarily of tricalcium silicate powder and a liquid containing calcium chloride as a setting accelerator [24]. It exhibits favorable biocompatibility, bioactivity, sealing ability, and dentin-like mechanical properties [24,25]. Consistent with these advantages, clinical studies and systematic reviews have reported favorable clinical and radiographic success rates for Biodentine pulpotomy in primary molars [26,27,28]. Its relatively short setting time and dentin-substitute properties make Biodentine particularly suitable for pulpotomy, as it can serve simultaneously as a pulp-covering material and a cavity-filling base before restoration [29]. During the hydration reaction, Biodentine forms calcium hydroxide, leading to the release of calcium and hydroxyl ions [30]. This alkaline and calcium-rich microenvironment contributes to its bioactivity, supports interfacial mineralization, and may promote the differentiation of pulp cells and reparative dentin formation [24,31,32,33]. In addition, Biodentine can form apatite deposits at the material–dentin interface, which may improve marginal sealing and reduce microleakage [34]. These properties provide a plausible biological basis for the stable postoperative response observed in the present cases. In several treated teeth, radiopaque bridge-like structures were observed beneath the Biodentine interface, which may be suggestive of reparative dentin formation. However, because radiographic findings alone cannot confirm the histological nature or continuity of the mineralized tissue, this observation should be interpreted cautiously.

The long-term success of pulpotomy is also influenced by the quality and timing of the final coronal seal [35]. A defective coronal restoration may permit microleakage and bacterial reinfection, eventually leading to late failure [16]. Several restorative materials have been used after pulpotomy, including resin composite, glass ionomer cement and SSC [36,37]. Resin-based restorations are esthetic, but their performance is technique-sensitive and may be affected by moisture control, polymerization shrinkage, marginal degradation, and the amount of remaining tooth structure [38,39]. Glass ionomer-based restorations release fluoride and may be useful for interim restoration and caries control [40]. However, their relatively limited wear resistance may restrict their use as a definitive restoration for extensively carious and pulp-treated primary molars. In contrast, SSCs provide full-coronal protection, reinforce structurally weakened primary molars, and reduce marginal breakdown by covering the remaining tooth structure [41]. Current pediatric restorative guidelines recommend SSCs for primary molars with extensive caries or multi-surface lesions, especially in high-caries-risk children [42]. Beyond the choice of restorative material, the timing of restoration is also important. In some studies, SSC restoration has been performed at a separate visit after pulp therapy [43,44]. Although this staged approach may allow postoperative observation, comprehensive dental treatment under general anesthesia generally aims to complete necessary procedures in a single session, thereby reducing repeated visits, additional behavior management, and potential re-exposure to anesthesia [45,46]. In the present case series, the short setting time and dentin-substitute properties of Biodentine allowed immediate SSC restoration during the general anesthetic session [47]. This approach provided a durable coronal seal for teeth with extensive structural loss.

General anesthesia was also an important clinical context in the present case series. Current behavior guidance recommendations indicate that general anesthesia may be appropriate for children who are unable to cooperate and for those requiring comprehensive dental care [48]. For young children with multiple deep carious lesions, this approach allows multiple procedures to be completed in a single visit and reduces reliance on patient cooperation. In the present case series, the pulpotomy procedure, the dentin-substitute properties of Biodentine, together with immediate SSC restoration, allowed pulp therapy and restoration to be completed during the same anesthetic session. This workflow may help avoid repeated visits and reduce the need for additional anesthetic exposure.

There are several limitations to this case series. First, this was a small, descriptive case series comprising five patients and 15 treated teeth. The small sample size and the clustering of multiple teeth within individual children make meaningful inferential statistical analysis inappropriate and limit the generalizability of the findings to all deeply carious primary molars. Second, the absence of a control group precludes conclusions regarding the comparative effectiveness of this protocol relative to other pulp therapy materials or treatment approaches. Third, Biodentine pulpotomy and immediate SSC restoration were performed as a combined protocol; therefore, the respective contributions of Biodentine and the SSC to the observed outcomes cannot be distinguished. Finally, the follow-up period was relatively short. Although the 1-year follow-up provides preliminary evidence of favorable short-term clinical and radiographic outcomes, the ideal endpoint for primary tooth therapy is long-term retention and function until physiological exfoliation. Therefore, longer follow-up is needed to evaluate the durability of this treatment approach.

Despite these limitations, this case series highlights the importance of integrating biological and restorative considerations when managing deeply carious primary molars under general anesthesia. In selected teeth, the absence of preoperative signs of irreversible pulpitis or apical periodontitis, together with bright-red pulp bleeding and hemostasis within 5 min, supported the decision to preserve the radicular pulp. Biodentine served as a bioactive pulp-covering and dentin-substitute material, while immediate SSC restoration provided definitive full-coronal protection during the anesthetic session.

## 4. Conclusions

Within the small descriptive case series, pulpotomy using Biodentine followed by immediate SSC restoration showed favorable short-term clinical and radiographic outcomes in selected deeply carious primary molars treated under general anesthesia. This single-session approach may provide a feasible option for preserving restorable primary molars when preoperative evaluation and intraoperative bleeding characteristics indicate a favorable radicular pulp status. Further studies with larger samples and longer follow-up until physiological exfoliation are needed to evaluate its long-term durability.

## Figures and Tables

**Table 1:** Patient-level treatment and follow-up timeline.

Patient	Included Teeth	Treatment Date	6-Month Follow-Up	Final Follow-Up
1	84, 85	21 June 2024	14 December 2024	24 August 2025
2	55, 74, 75	12 June 2024	14 December 2024	6 September 2025
3	54, 84, 85	26 June 2024	2 February 2025	19 July 2025
4	54, 55, 84	22 May 2024	14 December 2024	22 June 2025
5	54, 55, 84, 85	31 May 2024	14 December 2024	19 July 2025

## Data Availability

The data presented in this study are available on request from the corresponding author. The data are not publicly available due to privacy and ethical restrictions related to patient confidentiality.

## Abbreviations

The following abbreviations are used in this manuscript:

VPT	Vital pulp therapy
SSC	Stainless steel crown
